# The contribution of bacteriophages to the aetiology and treatment of the bacterial vaginosis syndrome

**DOI:** 10.12703/r/11-8

**Published:** 2022-04-19

**Authors:** Amaan Ali, Jan Stener Jørgensen, Ronald F Lamont

**Affiliations:** 1 St Bartholomew’s and The London School of Medicine and Dentistry, London, UK; 2 Department of Gynecology and Obstetrics, University of Southern Denmark, Institute of Clinical Research, Research Unit of Gynaecology and Obstetrics, Odense, Denmark; 3 Division of Surgery, University College London, Northwick Park Institute for Medical Research Campus, London, UK

**Keywords:** Bacterial vaginosis, Bacteriophages, Clindamycin, Lactobacilli, Metronidazole, Phage viruses, Sexual Transmission, Smoking

## Abstract

Bacteriophages are obligate intracellular viruses that parasitize bacteria, making use of the host biosynthetic machinery. Bacterial vaginosis (BV) causes serious adverse sequelae, such as sexually transmitted infections, seroconversion to HIV positivity, and preterm birth. The aetiology of BV is multifactorial, and the vaginal microbiota, the response to antibiotics, and the phenotypic outcomes differ between cases. The choice of antibiotics to treat BV depends on the clinician’s personal experience, which contributes to the poor outcome of BV treatment and high recurrence rate. In this review, we classify BV into two subtypes based on whether or not the BV case is sexually associated (potentially phage-related). An appropriate antibiotic can be selected on the basis of this BV-typing to optimise the short- and long-term effects of treatment. Not all *Lactobacillus* spp. are helpful or protective and some may sequestrate metronidazole, which mitigates its therapeutic efficacy. Phages, used therapeutically, could contribute to eubiosis by sparing beneficial species of *Lactobacilli*. However, *Lactobacilli* have an important role in maintaining vaginal eubiosis, so conventional wisdom has been that treatment of BV may benefit from metronidazole that conserves lactobacilli rather than clindamycin, which destroys lactobacilli. Furthermore, if the quality and quantity of vaginal lactobacilli are compromised by phage colonisation, as in the sexually transmitted subtype, eradication of lactobacilli with clindamycin followed by replacement by probiotics may be better therapeutically than metronidazole and reduce recurrence rates. Accordingly, the subtype of BV may provide a more scientific approach to antibiotic selection, which is absent in current clinical guidelines. We provide support for the role of bacteriophages in the aetiology, recurrence or failure to cure BV following treatment, through parasitic colonisation of lactobacilli that may be sexually transmitted and may be enhanced by other risk factors like smoking, a factor associated with BV.

## 1. Introduction

Bacterial phage viruses (bacteriophages) are obligate intracellular parasites that multiply by making use of the host biosynthetic machinery. Phages are extremely host-specific, so much so that phage typing has been used for many years to differentiate bacterial isolates, particularly food-borne pathogens, and in epidemiological studies to characterise outbreak-associated strains of bacteria^1–8^. Phages have also been used therapeutically for specific bacterial infections^9–14^, particularly in low- or middle-income countries because of the high cost of antibiotics^15^. Unlike with traditional antibiotics, this specificity of phages presents an attractive solution by targeting specific pathogenic bacteria. By preserving a eubiotic microbiota in niches such as the gut, eradication of the eubiotic gut microbiome which predisposes to secondary bacterial overgrowth with organisms such as *Clostridium difficile* and its sequelae, such as pseudomembranous colitis^16,17^, can be prevented. In addition, owing to multiple antibiotic resistances by opportunistic pathogens, other treatment approaches like therapeutic phages are attractive candidates because of their inability to invade eukaryotic human cells^18^. Furthermore, their specificity allows sparing of critically needed protective co-existent commensal non-pathogenic organisms that serve as part of the resident microbiota that make an important contribution to the long-term eubiosis of the vagina. This provides a safety margin, minimises adverse effects and amplifies nature’s response such that less frequent dosing is required compared with antibiotic use^19–25^. Phage therapy is a re-emerging modality to overcome bacterial resistance, and phages can be used naturally or synthetically as phage cocktails or as phage-antibiotic combinations. As they also treat biofilms with no side effects, phages show promising results for the treatment of bacterial infections as compared with antibiotics. According to the guidelines of the US Food and Drug Administration, phage therapy can be used in pharmaceuticals. Further funding is required by the pharmaceutical industry or governments for further investigation of bacteriophages in the management of bacterial vaginosis (BV)^26^.

Our aim is to emphasise that phage therapy may offer an advantage over antibiotics. This is not only because of their effectiveness against antibiotic-resistant bacterial pathogens but also because their specificity allows sparing of critically needed protective co-existent commensal non-pathogens that serve as the resident microbiota that influences the long-term health of the vagina. We aim to provide clinical guidance to select the appropriate antibiotics to treat BV on the basis of its subtype. According to whether phages are involved, we have divided BV into a number of subtypes that might influence the choice of treatment. According to our hypothesis, the first type is phage-related. In this type, normal vaginal lactobacilli are suppressed by infection by phages acquired sexually. To treat this type of BV, all phage-infected lactobacilli may need to be eradicated by a broad-spectrum antibiotic like clindamycin. The use of metronidazole that selectively eradicates anaerobes but not lactobacilli may not work as well. If phage-infected lactobacilli are not eradicated, these lactobacilli can serve as a reservoir to release more phages and BV can return after the treatment. This might explain why metronidazole has a low cure rate of only 54% in a clinical study (see end of Section 3.1) when these women still have residual vaginal lactobacilli.

The second part of our hypothesis is that some subtypes of BV are not phage-related. In this case, normal vaginal lactobacilli are suppressed by other causes, such as antibiotic overuse and douching. To treat this type of BV, indigenous lactobacilli are healthy and should not be cleared out by a broad-spectrum antibiotic like clindamycin. Metronidazole, which selectively kills anaerobes but not lactobacilli, should be used. This can allow the previously suppressed vaginal lactobacilli to multiply and repopulate the vaginal microbiota. There may also be a role for the use of probiotics to recolonize the vagina with a healthy microbiota.

### 1.1. Phage structure

**Figure 1.**
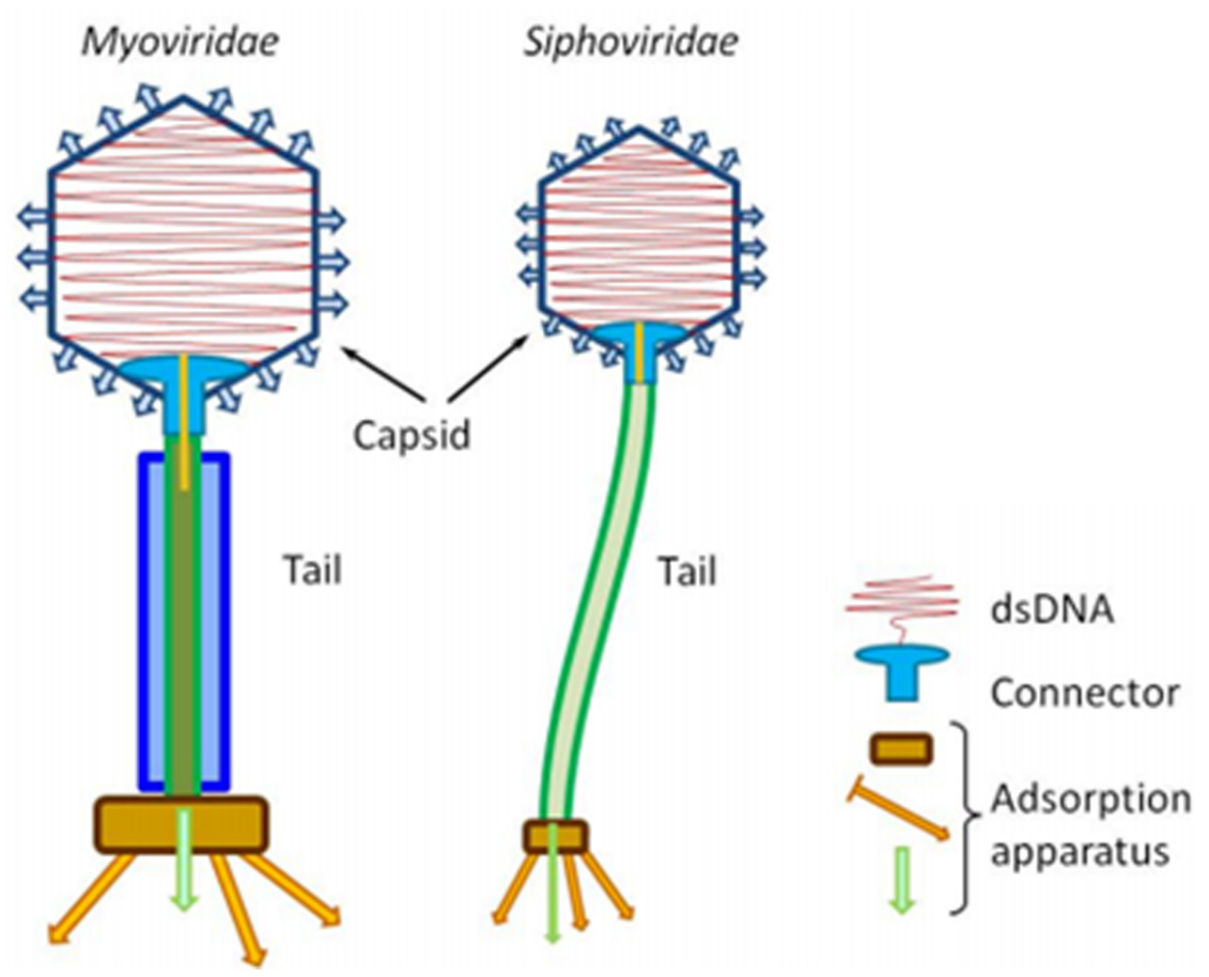
Structure of the phage virus.

All bacteriophages contain nucleic acids (either DNA or RNA but not both) and proteins. The nucleic acids contain some unusual bases, which render the phage DNA protected from nucleases, which are active against the host DNA during phage infection. The amount of DNA varies between strains; some code for only a few gene products, and other strains have sufficient DNA to code for 100 gene products. The proteins also differ in type, number and function but are involved mainly in the infection process and in protecting phage DNA from nucleases. The ultrastructure of bacteriophages differs between the many known strains^27^ and they vary in size from 24 to 200 nm. The head or capsid is commonly icosahedral (20-sided) but may be filamentous and is made up of one or more proteins that contain and protect the phage DNA/RNA. Many phage viruses have tails, which are hollow tubes through which nucleic acids pass to infect bacteria. Often the tail is surrounded by a contractile sheath and there is a base plate and tail fibres that, with other structures, facilitate binding to bacteria. There are two structural types of vaginal *Lactobacillus* phages. The most studied bacterial phage of *Myoviridae* is from the *Escherichia coli* phage T4. However, two types of phage structures have been reported among *Lactobacillus* phages: *Myoviridae* (with a contractile sheath) and *Siphoviridae* (without a contractile sheath) (Figure 1).

In the process of infecting host cells, tail fibres and analogous structures aid adsorption by binding with host-specific receptors on the bacterial cell wall. These structures may be lipopolysaccharides, pili or lipoproteins which the bacterium possesses for other functions but to which phage viruses have evolved to aid binding to the specific bacteria they infect. Although this adsorption is initially reversible, the process becomes irreversible through components of the base plate or other means. Subsequently, by contraction of the tail sheath or enzymatic degradation of the bacterial envelope, phage nucleic acids are injected into the bacterial cell. The nucleic acids are the only components of the phage virus to enter the bacterial cell; this is in contrast to animal viruses, where the whole virus becomes intracellular.

### 1.2. Life cycle of lytic phages

The multiplication cycle of bacteriophages depends on whether they are lytic (virulent) phages, which at the end of their life cycle kill the bacterial cell by lysis, or temperate (lysogenic) phages that remain quiescent within the bacterial cell. The genes of temperate phages are not transcribed, and the viral phage genome exists in a repressed state (prophage). The temperate viral phage nucleic acids integrate and replicate within the host bacterial DNA in daughter cells. The host cell that harbours the lysogenic prophage is not adversely affected by the lysogenic phage, which may persist indefinitely without any adverse effects to the host bacterium.

The life cycle of the lytic/virulent bacteriophage begins with the eclipse phase when the phage “eclipses” the host biosynthetic machinery by producing phage-specific mRNA and proteins. Early mRNA encodes for proteins, which promote phage DNA synthesis that blocks host DNA/RNA protein synthesis, and degrades host genetic material. Late mRNA encodes for structural proteins for phage multiplication and lysis of the bacterial cell during the subsequent accumulation, lysis and release phases. Up to 1000 phage viruses may be released by a single bacterium. Repression of the phage genome occurs through the synthesis of a phage-encoded repressor protein, which binds to an operator site on phage DNA and shuts off transcription of most phage genes except the repressor gene. Certain events can lead to the termination of lysogeny in temperate phages and the initiation and induction of a lytic cycle. This usually occurs following exposure of lysogenic bacteriophages to adverse conditions such as desiccation or exposure to ultraviolet light or ionising radiation or mutagenic chemicals. Under these circumstances, proteases that destroy the repressor protein are produced, leading to the expression of phage genes, reversal of the integration process and lytic multiplication. In all forms of life, transcription factors control gene expression^28^. Whether a bacteriophage enters a lytic or lysogenic cycle is determined by the concentration of two transcriptive repressor proteins. One switches off repressor synthesis and prevents lysogeny^29^. Environmental conditions that favour the production of the other transcriptive repressor protein leads to the lytic cycle.

### 1.3. Use in industry and medically related phage virus applications

Phages are abundant in the environment and are widely used in industry as biocontrol agents in food^30^ because of their specificity, their inability to alter the taste of food^31^ and their ability to tackle food-borne infections such as *E. coli*, *Listeria monocytogenes*, *Salmonella enterica* and *Campylobacter jejuni*^32,33^. Commercial ethanol fuel production rarely performs fermentations under aseptic conditions, and tanks are constantly contaminated with a wide variety of microbes (bacteria, fungi and wild yeast) in commercial biorefineries and reduce ethanol production with a costly shutdown of production. In addition to competing for nutrients and substrates with fermenting yeast, bacterial contaminants produce undesirable organic acids such as acetic and lactic acids that inhibit yeast growth, resulting in decreased yields of ethanol and product spoilage. Owing to their antibacterial activity, phages are considered alternatives to antibiotics and are useful alternatives to potentially resistant antibiotics during ethanol fuel fermentation^34^. Phage virus therapy for the treatment of BV is being developed. Although this technology uses phage endolysin rather than a *Lactobacillus* phage to kill *Gardnerella*, the basic concept of using phages to treat BV is important to the central theme of this article^35^.

## 2. The bacterial vaginosis syndrome

**Figure 2.**
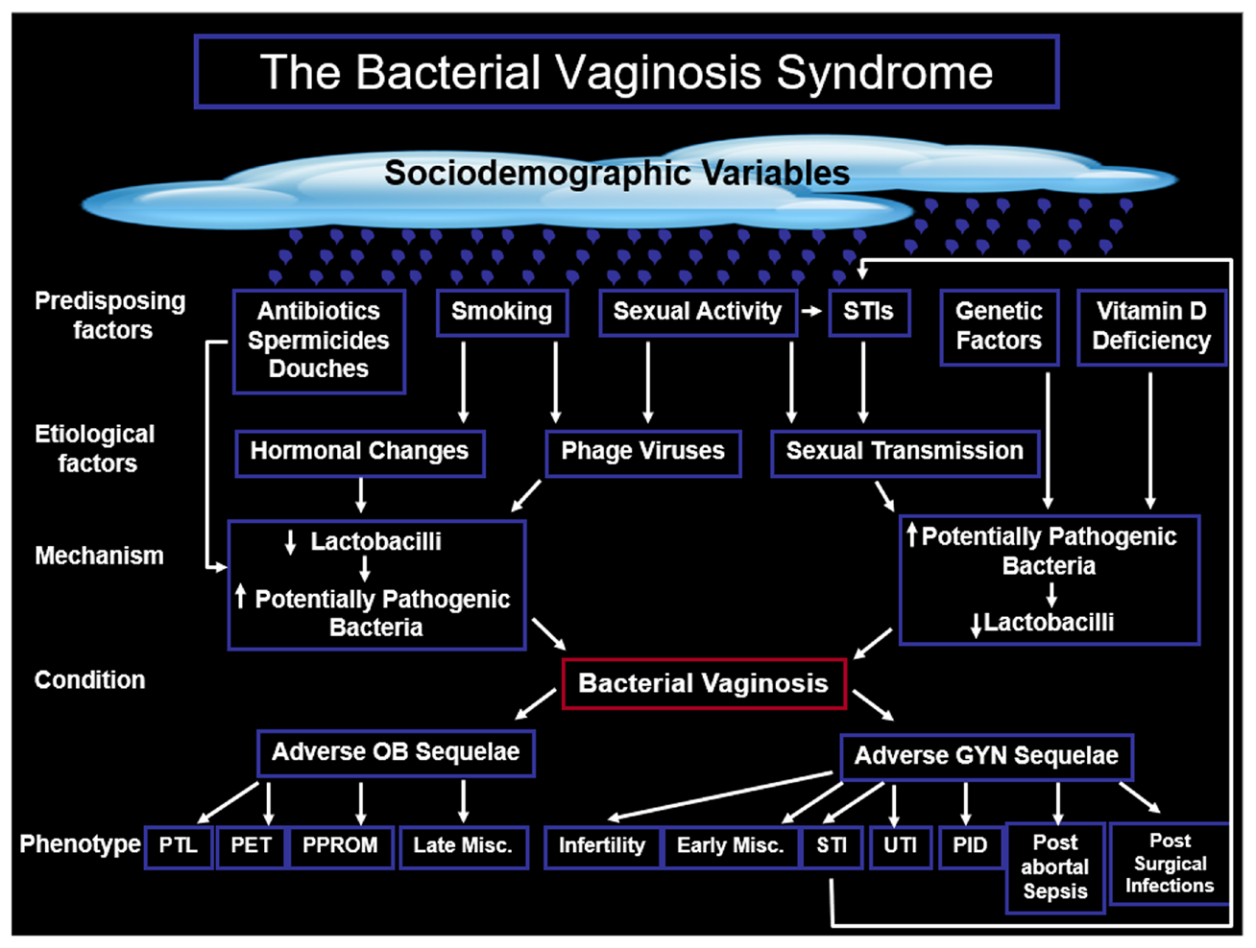
The proposed interaction among the mechanisms, aetiology, predisposing factors, and phenotypic outcomes in obstetrics and gynaecology of the bacterial vaginosis syndrome. GYN, gynaecological; Misc, miscarriage; OB, obstetric; PET, pre-eclampsia/toxaemia; PID, pelvic inflammatory disease; PPROM, preterm prelabour rupture of the membranes; PTL, preterm labour; STI, sexually transmitted infection; UTI, urinary tract infection.

BV, the commonest cause of vaginal dysbiosis in high-income countries, is responsible for one-third of all vulvovaginal infectious morbidity. BV is associated with serious and potentially life-threatening morbidity in both obstetrics and gynaecology, and although the aetiology of BV remains to be fully elucidated, it is likely to be multifactorial (Figure 2). In 1955, Gardner and Dukes^36^ were convinced that they had discovered the mono-etiological agent (now known as *Gardnerella vaginalis*) that caused non-specific vaginitis (NSV), which we now know as BV. We now know from molecular-based techniques that BV compared with vaginal eubiosis is associated with a significant diversity of other organisms that probably act symbiotically or synergistically to provide nutrients and other services that maintain the potentially pathogenic organisms that cause BV and other forms of dysbiosis^37^. In other words, “it takes two to tango”. Whether the symbiotic or synergistic diversity associated with BV begins with a reduction in the quality or quantity of lactobacilli (or both) followed by at least a 1000-fold increase in the number of other organisms, or vice versa, is unclear. BV is a treatable condition with high rates of cure or improvement, and a number of effective treatments are available. However, as many as 50% of women with BV experience recurrence within 6 to 12 months of treatment^38^. Alternative non-antibiotic options, such as probiotic products containing lactobacilli, lactic acid, sucrose gel, combination products with estriol, and supplementation of antibiotics, are available and show some promise^39^.

We do not feel that lysogenic bacteriophages are the primary cause of BV, but there is undoubted evidence of sexual transmission of both BV and phages, so we postulate that this contributes to recurrence or relapse of BV (see Section 3.2). We also accept that not all *Lactobacillus* spp. are helpful or protective and may sequestrate metronidazole, which mitigates its therapeutic efficacy, and that other phage viruses, as a therapeutic measure, could contribute to eubiosis by sparing beneficial species of *Lactobacilli*. However, species of *Lactobacilli* that provide species-specific benefits to support vaginal eubiosis can be parasitised by bacteriophages^40^. We speculate on the possibility that lysogenic phages contribute to the vaginal dysbiosis caused by BV by inducing a reduction in the quality and quantity of vaginal lactobacilli. This in turn may influence the choice of antibiotics and other options to treat BV and may explain the high recurrence of BV and the subsequent adverse sequelae.

The genus *Lactobacillus* underwent a major taxonomic reclassification in 2020. Before March 2020, the genus *Lactobacillus* comprised more than 260 phylogenetically, ecologically, and metabolically diverse species. In March 2020, the genus *Lactobacillus* was reclassified into 25 genera, which included 23 novel genera, which better reflected the phylogenetic heterogeneity of *Lactobacillus* clades. *Lactobacilli* that colonise the vagina, such as *L. crispatus*, *L. gasseri* and *L. jensenii*, are still within the genus *Lactobacillus*, but *L. vaginalis*, *L. reuteri* and *L. rhamnosus* (contained in many vaginal probiotic preparations) were reclassified as part of the *Limosilactobacillus* genus^41^.

### 2.1. Bacterial vaginosis as a sexually transmitted infection

Gardner and Dukes (1955)^36^ and Gardner in his 25-year review (1980)^42^ were in no doubt that BV (then known as *Gardnerella vaginitis*) was sexually transmitted. Without a doubt, the BV syndrome is sexually associated; it is increased in women with (i) early sexual debut, (ii) a greater number of lifetime sexual partners and (iii) the introduction of a new sexual partner. Increasingly, the epidemiology of some subtypes of BV supports sexual transmission^43^. However, whether BV is caused by a primary pathogen or a synergistic consortium of microorganisms that are sexually transmitted remains unclear^44^. It has been proposed that BV is initiated by sexual transmission of *G. vaginalis*^45,46^, which has the appropriate virulence factors to adhere to host epithelium, create a biofilm community, and successfully compete with lactobacilli for numerical dominance in the vagina.

### 2.2. Bacterial vaginosis not associated with sexual transmission

Opponents of the proposal that BV is a sexually transmitted disease point out that *G. vaginalis* can be found in women before sexual debut and in sexually active women with a healthy vaginal microbiota and that colonisation with *G. vaginalis* does not always lead to BV^47^ but this has been challenged^48^. There is some evidence in the form of self-reported surveys of confirmed BV in adolescents prior to self-reported sexual debut and BV incidence has been correlated to other risk factors for BV, such as regular vaginal washing^49^. However, these studies are small, and the self-reporting nature of such studies risks recall bias. In one study, further questioning of women self-reporting virginity identified a strong link between BV and non-penetrative sexual activity (*P* = 0.02) which feasibly could have led to sexually transmitted *Gardnerella*^50^. This is in keeping with the higher prevalence of BV in women who have sex with women, which was used historically to support the theory that BV was not sexually transmitted. Molecular-based studies using polymerase chain reaction fingerprinting demonstrated that among 31 female sex couples who were monogamous for more than 3 months, the distribution of genital *Lactobacilli* strains was identical in both partners in 77% of couples. The use of shared sex toys was postulated as one explanation^51^. Recent research into the pathogenesis of BV has determined the existence of a number of different species within the *Gardnerella* genus. It may be that healthy women are colonised by non-pathogenic species of *Gardnerella* but that virulent strains are involved in the development of BV^45^ (see Section 3.2).

## 3. The role of phages in the aetiology of bacterial vaginosis

Because of their putative health value, *Lactobacilli* are widely used in the dairy industry as starter cultures to ferment milk into yogurt and also as an additive to milk. By 2009, over 230 *Lactobacillus* phage viruses had been identified^52^ from various sources such as dairy products, sausages, human intestines and sewage^53–58^. Viable *Lactobacilli* may inhibit food-borne and gastrointestinal pathogens by producing lactic acid, hydrogen peroxide (H_2_O_2_) and other antimicrobial substances^59–62^. Accordingly, yogurt and acidophilus milk have been considered as having the potential for healthy probiotic diets^63^, although their effectiveness has been questioned^64^. The majority of clinical trials addressing vaginal probiotic use for the prevention of BV have demonstrated clinical efficacy. They demonstrate comparable efficacy of intermittent oral metronidazole treatment with a 2-month course of probiotic containing multiple strains of *Lactobacilli* in BV prevention as well as reducing the risk of having a dysbiotic vaginal microbiota (low abundance of *Lactobacilli* and high abundance and diversity of BV-associated anaerobes) on follow-up^65^. This warrants further large-scale studies into vaginal probiotics as they may offer an alternative or an adjuvant to antibiotics in the prevention of BV and adverse effects on the microbiota of other body systems. Probiotics may also be used to address the global crisis of antibiotic resistance. The subject of probiotics for the treatment of BV will be the subject of a separate review.

One of the most frequent reasons for the failure of large-scale industrial fermentation is an attack by lytic bacteriophages. In a small and highly controversial study, one particular brand of yogurt which contained a strain from the *Lactobacillus acidophilus* complex colonised the vagina and was reportedly successful in treating vaginal yeast infection^66^. Unfortunately, the strain disappeared and is no longer used and no longer identifiable. Bearing in mind the unlikely event of the manufacturer deleting such a potentially profitable strain, investigators postulated that the disappearance of the strain may have been due to natural causes, such as a lytic phage outbreak in which a virulent phage was released by a successor strain that eradicated its predecessor^53^. We introduce this study and the subsequent research to support our hypothesis that phage viruses may parasitise and reduce the quality or quantity of eubiotic species of *Lactobacilli* that may result in BV that is recurrent or resistant to treatment.

To test this hypothesis and to evaluate the stability of dairy *L. acidophilus* cultures, 20 yogurts and two acidophilus milks, commercially available in the USA, were purchased from local food markets and tested for the production of bacteriophages and bacteriocins (non-viable, non-DNA/RNA products or peptides that inhibit the growth of bacteria)^53^. From these purchases, 38 *Lactobacillus* strains were isolated; 11 of these released phages, whereas strains from most of the remaining products released bacteriocins. The authors concluded that such *Lactobacillus* probiotic products may be unstable or unsafe because they could be inhibited by phages or bacteriocins or because they release them to inhibit the lactobacilli of other dairy products^53^. Following this observation and the knowledge that phage infection delays acid production by starter cultures of *Lactobacilli* in the production of salami sausage^57^ and the putatively important protective role of *Lactobacilli* in preventing BV, the same group employed these findings to test the role of phages in the development of abnormal genital tract flora^67–69^.

Meta RNA sequencing conducted on four vaginal samples (two with confirmed BV and two with a eubiotic vaginal microbiota) revealed several transcriptomic differences between *Lactobacillus iners* in a eubiotic and a dysbiotic vaginal microbiota due to BV^70^. *L. iners*, the most abundant species of *Lactobacillus*, is found in both the eubiotic vaginal microbiota and vaginal dysbiosis, suggesting an ability to adapt to its environment to ensure continued existence^37,71^. In the presence of BV, *L. iners* increases its expression of clustered regularly interspaced short palindromic repeats (CRISPR), anti-bacteriophage genes that encode for proteins that suggest a response to phage attack in BV.

### 3.1. Isolation of phages from human vaginal *Lactobacilli*

It has been postulated that phage-mediated lysis of vaginal *Lactobacilli* might result in a profound decrease in the quantity of *Lactobacilli* and subsequent overgrowth of anaerobic bacteria. In an *in vitro* study of the vaginal secretions from 39 women of reproductive age, 12 out of 20 women with vaginal infections (16 with BV and four with vulvovaginal candidiasis [VVC]) had no *Lactobacilli*. In the remaining eight infected samples and the 19 samples from healthy women, 37 *Lactobacillus* strains were isolated. From these, seven temperate phages were identified. The rate of phage detection was less in healthy women and higher in those women with BV or VVC. The phages detected could infect vaginal *Lactobacilli* from the same women or those from different women, which has implications on the possible role of sexual transmission of phages. The authors also reported that phages isolated from a human intestinal strain of *Lactobacillus* were able to lyse some vaginal *Lactobacilli*, leading them to postulate that some of the vaginal phages may be derived from the lower gastrointestinal tract^68^.

In a follow-up experiment, similar to the one already described^53^, the ability of commercially available *Lactobacillus* products in the USA (and one vaginal douche mix) to produce phages and bacteriocins and their effect, this time on vaginal^69^ rather than dairy^53^ lactobacilli, was analysed*.* Forty-three strains or isolates of *Lactobacillus* were detected in the 26 commercially available products. Of these, 11 (obtained only from yogurt) released phages and seven of these inhibited vaginal *Lactobacilli.* The authors postulated that such widely available dairy products containing phages and bacteriocins might be involved in the pathogenesis of BV. From the same group, in a comparative study of vaginal *Lactobacilli*, the prevalent morphology, host range and DNA homology of phages isolated from American and Turkish women were examined. In total, 209 strains of vaginal *Lactobacillus* were isolated; of these, 67 phages, all of which were infective against *Lactobacilli* from the women of both countries, were isolated. The host ranges of most phages, including multiple *Lactobacillus* species, were broad. Though temperate, the phages were able to cause lytic infection in various strains of *Lactobacillus.* Overall, about 50% of lysogenic *Lactobacilli* were isolated from women with BV as opposed to about 30% in women without BV (*P* <0.05)^67^.

*L. crispatus* is considered the most protective species of *Lactobacillus* against BV-associated anaerobes; one study demonstrated a five-fold reduction in the conversion from normal to abnormal microbial flora in pregnant women with *L. crispatus* colonisation^72^. This is thought to be due to its secretion of H_2_O_2_ and lactic acid, including the ratio of the l- and d-isomers of lactic acid, and whether the lactic acid molecules exist as protonated (non-dissociated H^*+*^, neutrally charged ion) or a lactate anion (dissociated H^*+*^, negatively charged ion). The protonated form of lactic acid (which predominates at a pH <3.9) has antimicrobial and immunomodulatory properties compared with the lactate anion, which has no bactericidal or virucidal activity^73^. *L. crispatus* also has the ability to inhibit *G. vaginalis* adhesion to vaginal epithelium^74^.

A study comparing *L. crispatus* isolates in four BV-positive vaginal samples and four normal lactobacillus-dominated vaginal samples identified the presence of prophages within *L. crispatus* in both communities, but there was no correlation between the presence of BV with prophage induction capability and bacterial cell lysis on the administration of the DNA cross-linker mitomycin C^75^. Despite this, the authors conceded that phages may still influence the numbers of *L. crispatus* and this may not be explained solely by their inducibility. All of the strains of *L. crispatus* identified in the study were found to contain mobile elements, including phage genes and CRISPR repeats, and most strains contained phages that were inducible, suggesting prior exposure to phages. The CRISPR system, found in half of all bacterial species, is known to provide both adaptive and innate immunity against phages, similar to the immune system in humans^76^. The adaptive component to the CRISPR system can be seen in the observation that protein translation is guided by phage DNA which is incorporated into the bacterial genome in the form of spacers, allowing CRISPR RNA and subsequent cleavage proteins that specifically target and degrade phage DNA to be created^76^. The authors suggested that this past phage exposure could affect the stability of the strains in the face of a new type of phage or introduction of a phage induction factor, such as benzo-(a)-pyrene-dio-epoxide (BPDE) found in tobacco products, and that a combination of strain-specific genetic and environmental factors may influence lactobacillus numbers in BV.

A recent vaginal microbiome analysis of 48 women undergoing *in vitro* fertilisation for male factor infertility was conducted^77^. A comparison of BV-positive and BV-negative samples revealed statistically significant viral β-diversity, indicating a different viral community in BV-positive and -negative samples. A clear co-abundance pattern was identified between bacterial and phage populations. This permitted analysis of the virome and prediction of the bacterial community state type together with the presence of BV-associated pathogens or BV protective bacteria. BV-negative samples had a greater abundance of phages targeting *Lactobacillus* spp. than BV-positive samples. A high anaerobic bacterial abundance was negatively associated with the presence of *Lactobacillus*-infecting phages. This suggests that a low abundance of *Lactobacillus* results in fewer specific *Lactobacillus* phages and vice versa with a low abundance of BV-associated pathogens and their associated phages. The authors suggested that it is not prophage induction that is responsible for the depletion of *Lactobacilli* populations but rather the introduction of new, external lytic phages possibly acquired from a sexual partner. Alternatively, this may be due to the absence of protective phages in the vaginal mucosa against BV-associated pathogens facilitating colonisation of BV-associated pathogens and subsequent vaginal dysbiosis. This is due to highly diverse viral communities being associated with a smaller ratio of lysogenic phages than in less diverse viral communities in the study^77^. The presence of phages specific to *Lactobacillus* in BV-negative samples could suggest a protective role of these phages in developing *Lactobacillus* strains that are more resilient against potential exposure to external lytic phages.

A 2018 meta-transcriptomic study comparing 31 patients with BV who responded to metronidazole with six non-responders with persistent BV post-metronidazole therapy identified upregulated anti-phage CRISPR genes in *G. vaginalis* isolates, indicating possible phage attack within the vaginal environment^78^. The presence of lytic phages in the vaginal environment may reduce the effectiveness of metronidazole as the drug conserves the *Lactobacillus* community, which may be a host for these phages, resulting in recurrence. This was corroborated in a study in which microscopy and rRNA sequencing analysis of the vaginal microbiome was conducted before and after treatment with a standard 500 mg, twice-daily oral course of metronidazole in Rwandan women with diagnosed BV^31^. This study demonstrated a surprisingly low cure rate of only 54% with metronidazole in BV and a preserved and (in many cases) increased population of *Lactobacilli*^79^.

### 3.2. Bacterial “phaginosis”?

Collectively, this series of studies demonstrates that phages released from the vaginal *Lactobacilli* of some women can infect the *Lactobacilli* of other women under *in vitro* conditions^56^. Because intestinal *Lactobacillus* strains lysed multiple vaginal *Lactobacillus* isolates^68^, it may be that the lower intestine is a reservoir for phages that infect vaginal lactobacilli through the faeco–urogenital route. Alternatively (or additionally), the route may be through sexual transmission. In an editorial, Blackwell^80^ found the concept of phages causing BV interesting but not in keeping with the possibility that the condition is sexually transmitted. This highlights the possibility that vaginal *Lactobacilli* have phages^67^ and that lactobacillus-derived phages from dairy products can lyse vaginal *Lactobacilli*^68^. However, she considered that it was possible that temperate, diet-acquired phages are induced to become lytic by some other factor related to sexual activity or alternatively that *Lactobacillus* phages are directly inoculated into the vagina from male or female partners^51,80^.

### 3.3. Phage viruses from the environment

Since BV is a common condition and fewer than 20% of vaginal *Lactobacilli* are sensitive to dairy phages and millions of women each day ingest various yogurt products without apparent adverse events, it may be that if phages can affect the population of vaginal *Lactobacilli* in humans *in vivo*, then additional sources of phages exist. Sewage may be a rich source of phages against human bacteria because human waste collects there and phages are widely distributed in foods and food processes^81–91^. In a study published as an abstract, of seven phages isolated from two water treatment plants, six were able to lyse human vaginal *Lactobacilli*^92^. Like the findings of other studies, this finding was observed *in vitro* and may not be applicable *in vivo* in humans, but the observations suggest the possibility that vaginal *Lactobacilli* are inhibited naturally by environmental phages.

### 3.4. Smoking and phage viruses

Further evidence to support Blackwell’s hypothesis^80^ came from a report that BPDE (see Section 3.1), the metabolite of a chemical carcinogen in cigarette smoke, could be found in the vaginal secretions of women who smoke^93^. In a study of four lysogenic vaginal *Lactobacilli* and two control *Lactobacilli* from yogurt and the intestine, a number of smoke chemicals and their metabolites were tested for their ability to induce lysogenesis from temperate phages. With increasing concentrations of BPDE, there was an increase in the frequency of phage release. The authors concluded that chemicals in cigarette smoke promoted phage virus induction in vaginal *Lactobacilli* and that these phages lysed other vaginal *Lactobacillus* strains. In common with other bacteria, *E. coli* damages the host DNA by BPDE^94,95^, which inactivates the λ-phage repressor, and subsequently, functional phages are formed and released.

Other tobacco products can be found in cervical secretions^96,97^ in association with cervical intraepithelial neoplasia^98^ and in the semen of smokers^99^. Women who lack vaginal *Lactobacilli* are more likely to be smokers^100^, but the nanogram levels of smoke chemicals secreted into the genital tract would not be sufficient to kill or inhibit *Lactobacilli* directly^96,97,101–103^. An analysis of the vaginal metabolomes of 17 smokers and 19 non-smokers revealed greater concentrations of nicotine breakdown products (cotinine and hydroxycotinine) in smokers^104^. There were also increased levels of biogenic amines known to cause vaginal malodour, which form part of the diagnostic criteria for BV^37,105^. They are also known to increase susceptibility to invasion by pathogenic organisms in smokers, particularly those who had a low *Lactobacillus* environment (community state type IV)^104^, who are 25 times more likely to be a smoker^106^. BV is three times more common in women who smoke^107^, and BV and smoking are linked by common sequelae such as preterm birth, low birthweight, preterm prelabour rupture of the membranes, and transmission of HIV^107–110^. Accordingly, it is possible that women who smoke, or those whose partners smoke, secrete or introduce, by sexual transmission, tobacco products like BPDE into the vagina^111,112^. This places them at a greater risk of BV by inducing endogenous or sexually acquired *Lactobacillus* phages to become lytic and subsequently destroy normal vaginal lactobacilli.

## 4. Discussion

The aetiology of the BV syndrome remains to be fully elucidated but is likely to be multifactorial (Figure 2). The change from normal flora to the polymicrobial flora of BV may be initiated through a primary decrease in *Lactobacilli* leading to a secondary increase in potentially pathogenic bacteria or vice versa*.* If the primary event is due to a reduction in the quantity and quality of *Lactobacilli*, it is possible that temperate, diet-acquired or sexually acquired phage virus is induced to become lytic and adversely affect the quality or quantity of *Lactobacilli* followed by a secondary increase in potentially pathogenic bacteria. Although most of the data were produced in *in vitro* studies and may not be directly applicable to humans *in vivo*, these findings may have implications on the choice of antibiotic for the treatment of BV. An agent like metronidazole conserves *Lactobacilli* but if phages infect these *Lactobacilli*, then BV may remain untreated or quickly recur. In contrast, clindamycin destroys *Lactobacilli* and if these bacteria are diseased with bacteriophages, this may be advantageous by allowing healthy *Lactobacilli* to recolonise the vagina naturally or through the use of selected probiotics based on eubiotic-producing species of vaginal *Lactobacilli* like *L. crispatus*^37,113,114^.

### 4.1. Antibiotic choice depending on bacterial vaginosis subtype

We suggest that cases of BV may be divided into four subtypes: (i) recurrent, (ii) resistant, (iii) related to sexual transmission in a patient who has multiple sexual partners, a new partner, or where her partner has other sexual partners and (iv) not related to sexual transmission. Subtypes (i), (ii) and (iii) may be phage-related, in which case the first choice of antibiotic may be clindamycin. If, as in subtype (iv), the woman has no sexual partners (or is monogamous) and/or her BV is related to other factors unrelated to sexual contact or is a “one-off” event, this subtype may be non-phage-related, in which case the first choice of antibiotic may be metronidazole. For such women who do not respond to metronidazole, some would suggest that the possibility of aerobic vaginitis^115^ should be considered, although others have expressed caution about this as a diagnosis^116^.

### 4.2. Metronidazole versus clindamycin for the treatment of bacterial vaginosis and for the prevention of infection-related preterm birth

Table 1 compares the efficacy and recurrence of BV following treatment by metronidazole or clindamycin. A number of studies with subsequent systematic reviews and meta-analyses to address the confusion about the use of metronidazole or clindamycin for the treatment of BV and the prevention of infection-related preterm birth were reviewed in 2015^117^.

**Table 1.** Antibiotic options for the treatment of bacterial vaginosis and the pros and cons of clindamycin and metronidazole according to route of administration.

	Oral metronidazole	Topical metronidazole	Oral clindamycin	Topical clindamycin
Advantages	Conservation of “good” eubiotic-producing lactobacillus strains, which may be advantageous in non-phage-related bacterial vaginosis (BV). Targeted anaerobic coverage may be effective for anaerobe- predominant BV whilst preserving lactobacilli flora.	Same as with oral metronidazole. Topical application may limit systemic side effects due to reduced bioavailability.	Broad spectrum resulting in complete eradication of all lactobacillus strains, which may confer an advantage in bacterial “phaginosis”^79^. May confer an advantage in aerobic vaginitis because of broad- spectrum action.	Same as with oral clindamycin. Intravaginal application may limit systemic side effects due to reduced bioavailability; no serious adverse events were seen in a large randomised controlled trial (RCT) using 2% vaginal clindamycin cream for BV^120^.
Disadvantages	Increased incidence of nausea and vomiting and metallic taste in comparison with clindamycin^121^. Conservation of potential phage-infected lactobacilli potentially predisposes to BV recurrence in phage-associated BV (bacterial “phaginosis”). Cure rate as low as 54.5% reported with one course^79^. Recurrence rate of 69%–80% reported within 12 months^122^. Potential limited impact in aerobic vaginitis.	Same as with oral metronidazole. Reduced clearance of spp. of *Leptotrichia*, *Sneathia* and fastidious Clostridia-like bacteria compared with oral metronidazole^123^.	Increased risk of *Clostridium difficile* colitis due to broad-spectrum action and gut dysbiosis. Increased incidence of diarrhoea compared with placebo. In South Africa, a recent RCT of 136 pregnant patients with BV showed a non-significant increased failure rate (10.4% vs. 13%, *P* = 0.6) with oral metronidazole compared with oral clindamycin^124^.	Intravaginal clindamycin in addition to oral metronidazole was no more effective than oral metronidazole alone in an RCT of 450 patients^120^.

Our view is that clindamycin is more beneficial than metronidazole in this respect. The reasons for this and the theory behind the conclusion will be the subject of a separate publication. In brief, this theory centres on the fact that BV is not a single entity but involves a number of subtypes with different aetiologies, different vaginal microbiota, different responses to different antibiotics, and different phenotypic outcomes depending on the host response to the specific microbiome and the local milieu it creates^118^. *In vitro*, metronidazole is inactive against BV-associated organisms such as *G. vaginalis* and *Atopobium vaginae*^37^, organisms that are increasingly incorporated into molecular-based diagnostic tests for BV^119^. However, metronidazole is effective *in vivo*, so this is likely to be due to one of two reasons: (i) the pharmacologically active metabolite of metronidazole or (in our view, more likely) (ii) an indirect effect on the symbiotic relationship between organisms associated with BV. The mechanism by which this latter mechanism might occur involves the elimination of anaerobic organisms, for which metronidazole is particularly efficient because it cuts off the synergistic supply of nutrients to BV-related organisms such as *G. vaginalis* and *A. vaginae*. Through this property, metronidazole will successfully eradicate the anaerobic-dominated subtype of BV. In contrast, if the dysbiotic vaginal microbiota is dominated by an abundance of organisms that are BV-associated but are not predominantly anaerobes, metronidazole may be inactive and clindamycin and other antibiotics may be more effective. This may have direct relevance to the role of phages, vaginal *Lactobacilli*, the response to antibiotics used to treat the condition, the risk of recurrence and ultimately the adverse phenotypic outcomes in obstetrics and gynaecology.

## 5. Conclusions

We encourage further research into the role of vaginal *Lactobacillus* phage colonisation and its association with vaginal eubiosis and dysbiosis, particularly dysbiosis due to BV and VVC and subsequent adverse sequelae in obstetrics and gynaecology. Based on the multitude of studies that suggest a link between phages and BV, further larger-scale, *in vivo* research studies are warranted. We hope this will elucidate the precise mechanisms of causation as well as the impact of various therapeutic strategies on phage populations and BV status and subsequent adverse outcomes in obstetrics and gynaecology.

## Key messages

 •     Vaginal dysbiosis in the form of BV is an important cause of adverse sequelae in obstetrics and gynaecology.
 •     BV is characterised by a reduction in the quantity or quality of eubiotic vaginal *Lactobacilli* (or both) and a more-than-1000-fold increase in potentially pathogenic organisms, many of which are anaerobes.
 •     The aetiology of BV has not yet been fully elucidated, and the microbiology, whether based on cultivation-dependent or molecular-based techniques, differs from case to case. As such, the response to antibiotics differs; 6-month recurrence rates are on the order of about 50%, and the phenotypic outcome is inconsistent.
 •     Phages that selectively parasitise bacteria (bacteriophages) can remain temperate (lysogenic) or may be activated through a number of mechanisms like chemicals in tobacco to become lytic, multiplying and destroying their bacterial host.
 •     Bacteriophages have been demonstrated to infect eubiotic vaginal bacterial lactobacilli through sexual and other modes of transmission.
 •     Our hypothesis is that bacteriophage infection of eubiotic vaginal bacterial lactobacilli may be involved in the aetiology and influence the rate of recurrence of BV. Accordingly, the choice of treatment that conserves healthy *Lactobacilli* or destroys unhealthy phage-infected *Lactobacilli* may need to be considered. Following the latter, natural re-colonisation may be anticipated with eubiotic vaginal bacterial *Lactobacilli* or accelerated by seeding with probiotic *Lactobacilli.*
 •     We feel that this may beneficially affect the adverse outcomes of obstetrics and gynaecology associated with BV.

## Abbreviations

BPDE, benzo-(a)-pyrene-dio-epoxide; BV, bacterial vaginosis; CRISPR, clustered regularly interspaced short palindromic repeats; H_2_O_2_, hydrogen peroxide; VVC, vulvovaginal candidiasis
